# Hereditary Fibrinogen Aα-Chain Amyloidosis in Asia: Clinical and Molecular Characteristics

**DOI:** 10.3390/ijms19010320

**Published:** 2018-01-22

**Authors:** Masahide Yazaki, Tsuneaki Yoshinaga, Yoshiki Sekijima, Fuyuki Kametani, Nobuo Okumura

**Affiliations:** 1 Institute for Biomedical Sciences, Shinshu University, Matsumoto 390-8621, Japan; sekijima@shinshu-u.ac.jp; 2 Department of Clinical Laboratory Medicine, Shinshu University School of Health Sciences, Matsumoto 390-8621, Japan; nobuoku@shinshu-u.ac.jp; 3 Department of Medicine (Neurology and Rheumatology), Shinshu University School of Medicine, Matsumoto 390-8621, Japan; kiccho828@gmail.com; 4 Tokyo Metropolitan Institute of Medical Science, Tokyo 156-8506, Japan; kametani-fy@igakuken.or.jp

**Keywords:** fibrinogen Aα-chain amyloidosis, hereditary renal amyloidosis, Ostertag-type amyloidosis, laser microdissection, proteomics analysis

## Abstract

Hereditary fibrinogen Aα-chain amyloidosis (Aα-chain amyloidosis) is a type of autosomal dominant systemic amyloidosis caused by mutations in *fibrinogen A*α*-chain* gene (*FGA*). Patients with Aα-chain amyloidosis have been mainly reported in Western countries but have been rarely reported in Asia, with only five patients with Aα-chain amyloidosis being reported in Korea, China, and Japan. Clinically, the most prominent manifestation in Asian patients with Aα-chain amyloidosis is progressive nephropathy caused by excessive amyloid deposition in the glomeruli, which is similar to that observed in patients with Aα-chain amyloidosis in Western countries. In molecular features in Asian Aα-chain amyloidosis, the most common variant, E526V, was found in only one Chinese kindred, and other four kindred each had a different variant, which have not been identified in other countries. These variants are located in the C-terminal region (amino acid residues 517–555) of mature Aα-chain, which was similar to that observed in patients with Aα-chain amyloidosis in other countries. The precise number of Asian patients with Aα-chain amyloidosis is unclear. However, patients with Aα-chain amyloidosis do exist in Asian countries, and the majority of these patients may be diagnosed with other types of systemic amyloidosis.

## 1. Introduction

Different types of hereditary systemic amyloidosis without peripheral neuropathy are often referred to as “Ostertag-type amyloidosis” in reference to Ostertag’s description in 1950 of a German family in which several members died due to renal amyloidosis [1,2]. At present, hereditary non-neuropathic renal amyloidosis (also referred to as Ostertag-type amyloidosis) comprises a group of diseases associated with the variants of apolipoprotein AI [3], fibrinogen Aα-chain [4], lysozyme [5], apolipoprotein AII [6], apolipoprotein C2 [7], and apolipoprotein C3 [8].

Hereditary fibrinogen Aα-chain amyloidosis (Aα-chain amyloidosis) is a systemic disease caused by the extracellular deposition of amyloid fibrils comprising fibrinogen Aα-chain variants induced by mutations in *fibrinogen A*α*-chain* gene (*FGA*) [4]. To date, 16 amyloidogenic *FGA* mutations have been identified [4,9,10,11,12,13,14,15,16] (Table 1). The majority of patients with Aα-chain amyloidosis have been reported in European countries and the Unites States [2,17]. Although Aα-chain amyloidosis has also been reported in some Asian patients [12,13,14], it is still recognized as a rare disease in Asia. Aα-chain amyloidosis was not reported in Japan until we recently identified a sporadic case of a patient who was initially diagnosed with primary AL amyloidosis [14]. This indicates that some patients with Aα-chain amyloidosis who have not been correctly diagnosed may exist in Asia because of the low recognition of the disease and clinical similarity of this disease to other types of amyloidosis. Here, we describe the clinical and genetic features of Aα-chain amyloidosis by paying special attention to Asian patients with Aα-chain amyloidosis, particularly the clinical and molecular characteristics of the first Japanese patient diagnosed with Aα-chain amyloidosis [14].

## 2. Fibrinogen Aα-Chain Amyloidosis

The first case of Aα-chain amyloidosis was described by Benson et al. [4] in 1993 in a Peruvian-Mexican population, segregating with a mutation in *FGA* as R554L. Aα-chain amyloidosis is characterized by excessive amyloid deposition preferentially in the renal glomeruli and clinically presents as proteinuria, hypertension, and azotemia [2]. Involvement of the liver and spleen may be observed in advanced cases of Aα-chain amyloidosis; however, excessive amyloid accumulation in the glomeruli is the most remarkable feature of this disease [2]. Moreover, amyloid deposits result in atherosclerosis or cardiomyopathy in some patients [17,18]. A previous study reported that clinical symptoms of Aα-chain amyloidosis usually begin from the fourth to the ninth decade of life and that the median time from the progression of proteinuria to end-stage renal disease (ESRD) is 4.6 years [13]. However, some patients develop Aα-chain amyloidosis symptoms from the first or the second decade of life [11,12]. Most patients with Aα-chain amyloidosis do not have any family history of renal disease or amyloidosis because of the variable penetrance of this disease [13]. Therefore, a sporadic case of Aα-chain amyloidosis may be frequently misdiagnosed as primary AL amyloidosis [19]. To date, 16 amyloidogenic *FGA* variants have been reported, of which seven are missense mutations and nine are deletion or insertion-deletion mutations (Table 1) [4,9,10,11,12,13,14,15,16]. Of the nine deletion or insertion-deletion mutations, eight result in an out-frameshift mutation. E526V is the most common variant detected in patients across the world [2,9,13,17,20,21] (Table 1). Most patients with Aα-chain amyloidosis are heterozygous for the E526V variant; however, a recent study reported a Portuguese patient who was homozygous for the E526V variant [22]. While a genotype-phenotype correlation has not been investigated in detail, all described amyloidogenic mutations have been found to be associated with renal amyloidosis [2,13]. In addition, patients with Aα-chain amyloidosis having frameshift variants seem to show earlier disease onset (median age, 30 years) than those with single amino acid substitutions (median age, 59 years) [15].

## 3. Aα-Chain Amyloidosis in Asia

Aα-chain amyloidosis is the leading cause of hereditary non-neuropathic systemic amyloidosis in Western countries [17]. However, only a few cases of Aα-chain amyloidosis have been reported in Asia [12,13,14,23]. In 2005, Kang et al. [12] reported a sporadic case of a patient with Aα-chain amyloidosis in Korea who showed very early onset of the disease (at the age of 7 years) and rapid deterioration of renal function. The patient developed ESRD within 2 years after the disease onset. Renal biopsy showed severe glomerular enlargement with excessive amyloid deposits. Moreover, this patient had a unique frameshift mutation (c.1606_1620del,1619_1620insCA) in *FGA*, which was the first insertion-deletion mutation to be reported in patients with Aα-chain amyloidosis. Later, Gillmore et al. [13] detected two novel variants, T538K and c.1632delT (a frameshift variant), in Chinese patients with Aα-chain amyloidosis. In contrast, Yao et al. [23] reported the case of a Chinese patient with Aα-chain amyloidosis who had the most common mutation, i.e., E526V, and who presented with proteinuria, hypertension, and renal failure.

## 4. The First Case of Aα-Chain Amyloidosis in Japan: Clinical and Molecular Features

**Figure 1:**
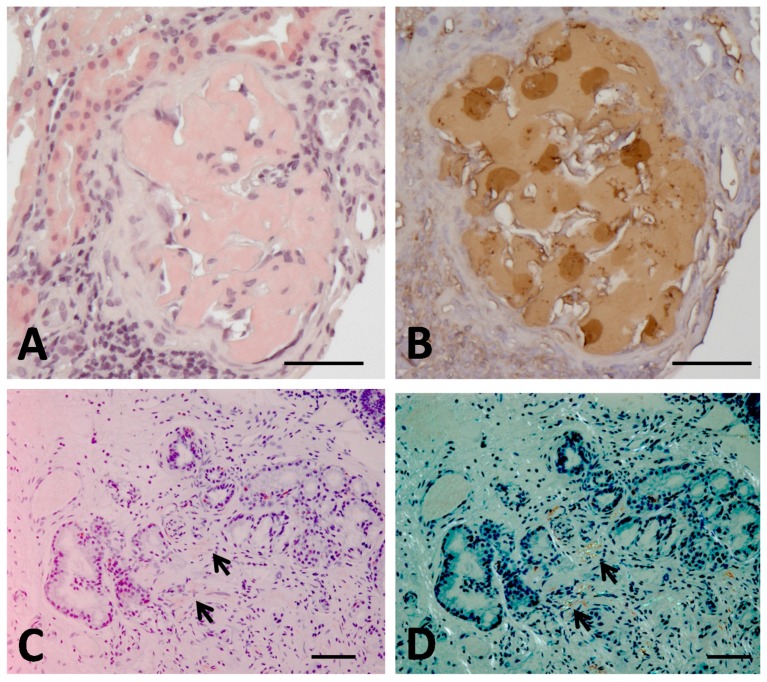
Findings of renal biopsy (**A**,**B**) and duodenal mucosal biopsy (**C**,**D**) in a Japanese Aα-chain amyloidosis patient [14]. (**A**,**C**) Congo red staining. (**B**) Immunohistochemical staining with anti-human fibrinogen antibody. (**D**) Congo red staining under polarized light. Renal biopsy at the age of 32 years shows heavy amyloid deposits mainly in glomerulus with almost complete obliteration of the normal glomerular architecture (**A**). The positive staining is observed in immunohistochemistry (**B**). Duodenal mucosal biopsy at the age of 40 years demonstrates a small amount of amyloid deposits (arrows, (**C**)), showing typical apple-green birefringence under polarized light (arrows, (**D**)). Bars = 50 μm.

**Figure 2:**
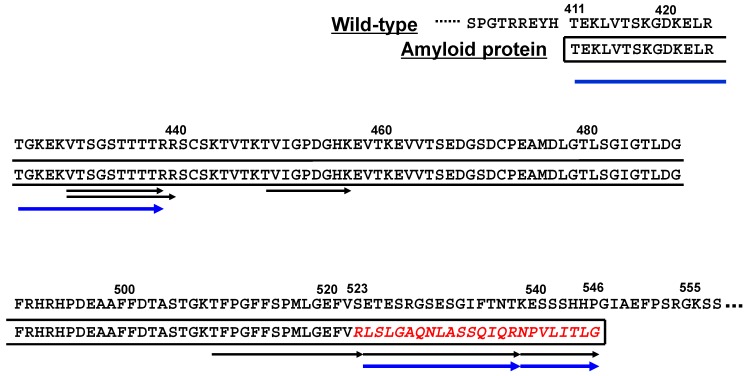
Putative primary structure of amyloid fibril protein in a Japanese Aα-chain amyloidosis patient [14]. Amyloid protein in this patient is assumed to be composed of both wild-type sequence (residues 411–522) and an additional fragment (red italic letters) induced by the frameshift variant (residues 523–546). Black arrows denote tryptic peptides detected at laser microdissection (LMD) isolation of glomerular amyloid, and blue arrows denote Arg-C peptides detected at in-gel digestion on SDS-PAGE analysis of duodenal amyloid.

In Japan, the vast majority (over 90%) of patients with hereditary systemic amyloidosis are transthyretin-related familial amyloid polyneuropathy (hereditary ATTR amyloidosis). More than 300 patients are assumed to exist in Japan, and those patients usually present with severe peripheral and autonomic neuropathy, cardiomyopathy gastroenteropathy, and/or oculopathy. However, no patient with hereditary non-neuropathic renal amyloidosis (Ostertag-type amyloidosis) had been reported in Japan until we reported the sporadic case of a patient with Aα-chain amyloidosis having a novel frameshift variant (4899_4902delAGTG) in 2015 [14]. This patient was a 40-year-old woman with rapidly deteriorating nephropathy [14]. The patient developed proteinuria at the age of 32 years, and renal biopsy showed excessive amyloid deposits in her glomeruli (Figure 1A,B). The patient did not have any overt signs of neuropathy, cardiomyopathy, oculopathy, or gastroenteropathy; however, gastroduodenal biopsy showed small amount of amyloid accumulation in the duodenal mucosa (Figure 1C,D). The plasma fibrinogen level of the patient was within the normal range (254 mg/dL; normal range, 180–350 mg/dL), and she did not show coagulopathy. Abdominal fat pad biopsy and liver biopsy did not show the presence of amyloid deposits (data not shown). Initially, the patient was diagnosed with primary AL amyloidosis. However, her renal function deteriorated rapidly despite treatment with combined chemotherapies, and she developed ESRD at 18 months after the detection of proteinuria. Eight years after the onset, a definite diagnosis of Aα-chain amyloidosis was made by proteomics analysis of glomerular amyloid on renal sample biopsied at 32 years, using laser microdissection (LMD) and liquid chromatography tandem mass spectrometry (LC-MS/MS) [14]. Proteomics analysis detected several tryptic peptides in accordance with the C-terminal region of Aα-chain (Figure 2). In addition, proteomics analysis detected two tryptic peptides, namely, LSLGAQNLASSQIQR and NPVLITLG, that corresponded to the previously described sequences of the C-terminal region of a fibrinogen variant (position 525–547) induced by a frameshift mutation (4904delG [10]) (Figure 2). Proteomics analysis did not detect a wild-type Aα-chain sequence after position 523, indicating that amyloid fibrils in our patient were only composed of the variant Aα-chain fragment. Moreover, proteomics analysis did not detect other tryptic peptides before position 430, suggesting that the N-terminus of the amyloid protein was present around position 430 (Figure 2). To obtain more information on the N-terminus of the amyloid protein, we biochemically investigated the amyloid fibril protein isolated from frozen duodenal mucosa samples by performing methods reported previously [24] and detected several peptides generated by Arg-C peptidase (Figure 2). We also detected a fragment starting with the amino acid residue Thr at position 411, but could not detect peptides before position 410. Therefore, we assumed that Thr411 was the N-terminus and that the amyloid protein was composed of Aα-chain fragments with wild-type (positions 411–522) and abnormal sequences (position 523–546) (Figure 2). However, we did not detect any wild-type sequence after residue 523. This result indicated that the wild-type Aα-chain sequence did not contribute to amyloid formation in our patient, as that observed in another patient with 4897delT [11]. SDS-PAGE showed that the molecular weight of the amyloid fibril protein was approximately 12 kDa (unpublished observation).

## 5. Discussion

One of the most notable clinical features of Aα-chain amyloidosis in Asia is that it may still be a quite rare disease, as mentioned above, and only a few Asian patients have been found to date, solely in East Asian countries [12,13,14,23]. Nephropathy is the main clinical manifestation in Asian patients with Aα-chain amyloidosis, which is similar to that observed in patients with Aα-chain amyloidosis in other countries. Moreover, Asian and Western patients with Aα-chain amyloidosis show similar renal pathological findings, including excessive amyloid deposits in the glomeruli [12,14]. Genetically, the five Asian patients with Aα-chain amyloidosis had five different genetic mutations, of which four were novel (Table 1). E526V, the most common variant in patients with Aα-chain amyloidosis in Western countries, was detected in only one Chinese patient with Aα-chain amyloidosis [23]. The other four mutations detected in the remaining Asian patients with Aα-chain amyloidosis have not been reported in patients with Aα-chain amyloidosis in other countries to date.

**Figure 3:**
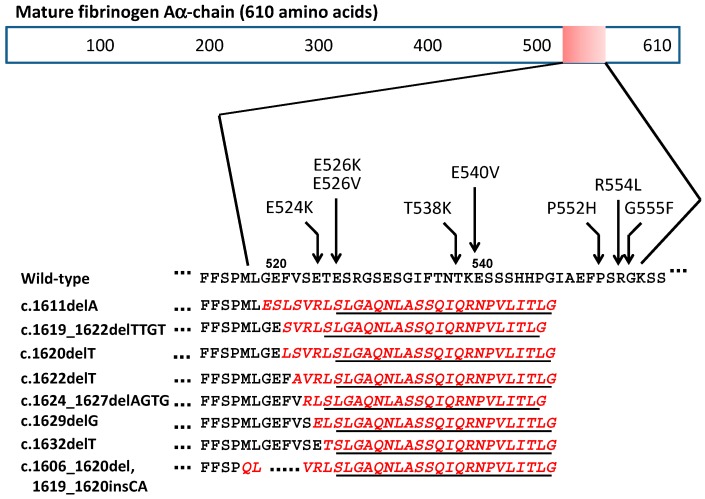
The location of 16 amyloidogenic *FGA* variants. All amyloidogenic variants crowd in a quite limited portion from residue 517 to 555 of the mature Aα-chain. In eight frameshift variants, the C-terminal sequences resulted from frameshift variants (red italic letters) bear a close resemblance to each other, and the C-terminal peptides consisting of 24 amino acids (underline) are identical.

Fibrinogen is a 340-kDa plasma glycoprotein that plays a major role in blood coagulation by converting to fibrin [25,26]. It is composed of two identical subunits, each of which contains an Aα-, Bβ-, and γ-chain and is produced by hepatocytes. Mature Aα-chain contains 610 amino acids and has a molecular weight of approximately 66 kDa [27]. The detailed pathomechanism underlying amyloid formation from the Aα-chain remains unclear. Interestingly, all amyloidogenic variants, including the eight frameshift mutations, are present in a specific region of the Aα-chain from residues 517 to 555 (Figure 3). These data clearly suggest that the location of *FGA* mutations is a key factor in amyloid formation. However, because five single amino acid substitutions (Available online: www.geht.org/databaseang/fibrinogen/), such as R554C [28] and R554H [15] at position 517–555, do not induce amyloid formation, it is likely that amyloid formation strongly depends on both the location of the variants and the type of replaced amino acids. In addition, of particular interest in the eight frameshift variants is that the C-terminal sequences resulting from all mutations bear a close resemblance to each other (Figure 3). In fact, C-terminal peptides containing 24 amino acids (SLGAQNL.....VLITLG) are identical among all of the frameshift variants, suggesting that this common sequence may be essential for amyloid formation. A recent study reported the importance of the C-terminal sequence composed of five amino acids (VLITL) in amyloidogenesis [16]. This common sequence may be attributed to the number of deleted nucleotides explained by arithmetical progression (1 + 3[n − 1]). In addition, it seems to be important that the location of nucleotide deletion should be after TAG within Leu518 (TTA) and Gly519 (GGA), or all nucleotides at position 518 and 519 are deleted, according to the mutation reported by Kang et al. [12]. Indeed, one nucleotide deletion (delC) at position Ala499 (GCC) generated a premature stop codon (TAG) at position 518, and this mutation was non-amyloidogenic [29]. On the other hand, in amyloid proteins associated with frameshift mutations, the length of the wild-type amino acid sequence in the N-terminus seems to vary in each case. In our patient, the presumable start point was Thr411 and the amyloid protein was assumed to contain 136 amino acids (411–546), with a molecular weight of approximately 12 kDa (Figure 2). In contrast, the putative starting point in an amyloid protein associated with 4897delT was assumed to be around Ala499, and its molecular weight was found to be 5 kDa [11]. Therefore, the production of a fragment containing an appropriate length of the wild-type Aα-chain sequence and an additional 24 common C-terminal residues seems to be important for amyloid formation. It was assumed that the variant Aα-chain containing 546 amino acid residues, which was detected in our patient with a 4-bp deletion [14], was more unstable and could be more easily degraded than the normal Aα-chain, as reported in other frameshift variants [10]. However, because no amyloid deposits were seen within or outside hepatocytes in our patient, it is possible that the variant Aα-chain was secreted by hepatocytes without intracellular aggregation. Therefore, at least in our patient, amyloid formation seemed to be strongly dependent on some proteolytic events after secretion from hepatocytes. In the variant Aα-chain with a single amino acid substitution (R554L), the amyloid protein consisted of a fragment containing variant Aα-chain residues 500–580 [4]. Serpell et al. [30] described the importance of residues 500–521 in amyloidogenesity because this portion was also present in a frameshift variant (4897delT) [11]. This portion was also detected in our patient (Figure 2). This suggests that an amyloidogenic fragment of an appropriate length for fibril formation generated from a variant Aα-chain through an aberrant proteolytic event is important for amyloid formation. However, mechanisms underlying the generation of amyloidogenic fragments or their escape from further processing are unclear, and further in vivo or in vitro investigations should be performed to address these questions.

Thus, more patients with Aα-chain amyloidosis may be present in Asia. However, the majority of these patients may have been misdiagnosed with other types of systemic amyloidosis. Asian and European patients with Aα-chain amyloidosis do not show remarkable differences with respect to clinical manifestations; however, the most prevalent genetic variant E526V in European patients may not be common in Asian patients. Recently, several effective therapies have been established for treating different types of systemic amyloidosis; however, the therapeutic strategy for treating each type of amyloidosis is different [13,17,31,32,33,34,35,36,37,38,39,40,41,42] (Table 2), highlighting the importance of the correct diagnosis of the disease at an early stage. Moreover, the possibility of Aα-chain amyloidosis should always be considered in sporadic cases of amyloid nephropathy, even in Asian countries. In our patient, proteomic analysis by performing LMD and LC-MS/MS not only helped in making the correct diagnosis, but also helped in obtaining information on the whole sequence of the amyloid fibril protein by using small sections of an old formalin-fixed, paraffin-embedded tissue sample obtained from the patient [14].

## 6. Conclusions

Clinical pictures in Asian Aα-chain amyloidosis patients are almost similar to those in Western counties. While the features of genotype in *FGA* may be different between Asian and European patients, it is common that all amyloidogenic variants are located in the C-terminal region (amino acid residues 517–555) of mature Aα-chain. The precise number of Asian patients with Aα-chain amyloidosis is unclear. However, patients with Aα-chain amyloidosis do exist in Asian countries, and the majority of these patients may be diagnosed with other types of systemic amyloidosis.

## Figures and Tables

**Table 1:** Sixteen amyloidogenic *FGA* mutations.

*FGA* Mutations	Protein Variants	Clinical Features	Geographic Origin/Ethnicity	References
**Missense Mutations**				
c.1627G > A	E524K	Nephropathy	Scottish	Rowczenio et al. [15]
c.1633G > A	E526K	Nephropathy	Russian	Rowczenio et al. [15]
c.1634A > T	E526V	Nephropathy	US, Europe, Brazil, China	Uemichi et al. [9]
c.1670C > A	T538K	Nephropathy	Chinese	Gillmore et al. [13]
c.1676A > T	E540V	Nephropathy	German	Gillmore et al. [13]
c.1712C > A	P552H	Nephropathy	Afro-Caribbean	Gillmore et al. [13]
c.1718G > T	R554L	Nephropathy	Mexico, Europe, US (Afro-American)	Benson et al. [4]
**Deletion Mutations**				
c.1611delA (4886delA)	G519EfsX548	Nephropathy	French	Rowczenio et al. [15]
c.1619_1622delTTGT (4894_4897delTTGT)	F521SfsX547	Nephropathy	North African	Rowczenio et al. [15]
c.1620delT (4895delT)	F521LfsX548	Nephropathy	French	Garnie et al. [16]
c.1622delT (4897delT)	V522AfsX548	Nephropathy	French	Hamidi et al. [11]
c.1624_1627delAGTG (4899_4902delAGTG )	S523RfsX547	Nephropathy	Japanese	Yazaki et al. [14]
c.1629delG (4904delG)	E524EfsX548	Nephropathy	US	Uemichi et al. [10]
c.1632delT (4907delT)	T525TfsX548	Nephropathy	Chinese	Gillmore et al. [13]
**Insertion-Deletion**				
c.1606_1620del, 1619_1620insCA (4881_4895del, 4894_4895insCA)	M517_F521del insQSfsX548	Nephropathy	Korean	Kang et al. [12]
c.1720_1721delGGinsTT (5445_5446delGGinsTT)	G555F	Nephropathy	Norwegian	Rowczenio et al. [15]

Number of amino acids denotes the position in mature fibrinogen Aα-chain.

**Table 2:** Therapeutic options in major systemic amyloidoses.

Types of Systemic Amyloidosis	Precursor Proteins	Therapeutic Options	References
Hereditary ATTR amyloidosis	Transthyretin	Liver transplant, tafamidis, diflunisal, gene therapies	[31,32,33,34,35]
Primary AL amyloidosis	Immunoglobulin light chain	Chemotherapies, stem cell transplantation	[36,37,38]
Reactive AA amyloidosis	Serum amyloid A (SAA)	Control of underlying diseases, biologic agents	[39,40]
Fibrinogen Aα-chain amyloidosis	Fibrinogen Aα-chain	Renal transplant, liver and renal transplant	[13,17]
Apolipoprotein A-I amyloidosis	Apolipoprotein A-I	Renal transplant, liver and renal transplant	[41]
Apolipoprotein A-II amyloidosis	Apolipoprotein A-II	Renal transplant	[42]

## Abbreviations

Aα-chain amyloidosis	Fibrinogen Aα-chain amyloidosis
ESRD	End-stage renal disease
LMD	Laser microdissection
LC-MS/MS	Liquid chromatography-tandem mass spectrometry
